# Application of Green Cobalt Nanoparticles in the Diet of Broiler Chickens to Improve Sustainable Production and Health

**DOI:** 10.1002/vms3.70508

**Published:** 2025-07-18

**Authors:** Fayiz M. Reda, Abdullah S. Alawam, Hemat K. Mahmoud, Mohamed T. El‐Saadony, Ayman S. Salah, Hassan A. Rudayni, Ahmed A. Allam, Karima El‐Naggar, Mahmoud Alagawany, Most Khairunnesa

**Affiliations:** ^1^ Poultry Department Faculty of Agriculture Zagazig University Zagazig Egypt; ^2^ Department of Biology College of Science Imam Mohammad Ibn Saud Islamic University (IMSIU) Riyadh Saudi Arabia; ^3^ Department of Animal Production Faculty of Agriculture Zagazig University Zagazig Egypt; ^4^ Department of Agricultural Microbiology Faculty of Agriculture Zagazig University Zagazig Egypt; ^5^ Department of Animal Nutrition and Clinical Nutrition Faculty of Veterinary Medicine New Valley University El‐Kharga Egypt; ^6^ Department of Nutrition and Veterinary Clinical Nutrition Faculty of Veterinary Medicine Alexandria University Alexandria Governorate Egypt; ^7^ Department of Dairy and Poultry Science Faculty of Veterinary Medicine and Animal Science Gazipur Agricultural University Gazipur Bangladesh

**Keywords:** blood, broiler, digestive enzymes, growth, nano cobalt

## Abstract

**Background:**

Nanoparticles (NPs), such as green cobalt NPs (CoNPs), are easier to pass through cell membranes in animals and interact rapidly with biological systems. Therefore, using green CoNPs is one of the recommendations for enhancing the bioavailability of cobalt, thereby improving its absorption.

**Objectives:**

The goal of this study was to explore the influences of biological nano‐cobalt (BNCo) as a feed supplement on broiler growth performance, haematology, blood chemistry, antioxidant activities, immunological status, digestive enzymes and carcass characteristics.

**Methods:**

A total of 300 Arbour Acre broiler chicks, all unsexed, were distributed into 5 treatment groups, each containing 60 chicks, at random. Five replications of each group were formed, each containing 12 chicks. The first group received a control diet free of BNCo, whereas the second, third, fourth and fifth groups were administered diets fortified with BNCo at concentrations of 100, 200, 300 and 400 ppm, respectively.

**Results:**

The results illustrated a significant increase in body weight (*p* < 0.001), body weight gain (*p* < 0.001) and improvement in feed conversion ratio (*p* < 0.01) in comparison to the control diet. BNCo supplementation significantly increased the percentage of immune organs, especially the spleen and thymus (*p* < 0.05, *p* < 0.01). Moreover, BNCo supplementation significantly increased haemoglobin (Hb) (*p* < 0.01), red blood cells (RBCs) (*p* < 0.05) and white blood cells (WBCs) (*p* < 0.05). In broiler chickens, BNCo supplementation increased serum levels of total protein, albumin and globulin significantly (*p* < 0.05) and reduced serum levels of aspartate aminotransferase (AST) activity (*p* < 0.001) and alanine aminotransaminase (ALT) (*p* < 0.01). The BNCo in feed supplementation raised blood levels of cobalt and markedly improved lipid markers, immunological and antioxidant status (*p* < 0.05). Furthermore, digestive enzymes were significantly boosted (*p* < 0.05) by BNCo treatments.

**Conclusion:**

The results indicated that BNCo supplementation at 200 ppm/kg diet produced the best overall performance, demonstrating its potential as an innovative additive for broiler diets.

## Graphical abstract

Nanoparticles, such as green cobalt nanoparticles, are easier to pass through cell membranes in animals and interact rapidly with biological systems. The results indicated that BNCo supplementation at 200 ppm/kg diet produced the best overall performance, demonstrating its potential as an innovative additive for broiler diets.

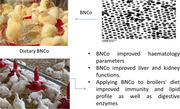

## 1 Introduction

The fields of animal and veterinary sciences are currently witnessing the advent of nano‐biotechnology for several beneficial applications, including nutritive, therapeutic and diagnostic (Patra et al. 2020; El‐Maddawy et al. 2022; AL‐Ruwad et al. 2024). Nanoparticles (NPs) of important minerals, sized between 1 and 100 nm, may provide a substitute for traditional elemental forms in animal nutrition (Mohamed et al. 2016; Swain et al. 2016; Scott et al. 2018; Kociova et al. 2020). It is posited that significantly reduced quantities of NPs will be sufficient to cover the elemental requirements of animals compared to bulk minerals (Abdollahi et al. 2020; Szuba‐Trznadel et al. 2021; Ouyang et al. 2021; Amr et al. 2025), thereby mitigating the environmental effects associated with high concentrations of inorganic salts (Vijayakumar et al. 2014; Ouyang et al. 2021). Decreased mineral concentrations in animal feeds may lead to reduced feed expenses. Furthermore, the characteristics of reduced size, enhanced similarity, considerable surface area and increased physical activity of nano‐forms of minerals may boost bioavailability in the gastrointestinal tract (GIT) of the animals (Hill et al. 2017; Youssef et al. 2019; Hidayat et al. 2021). Animals may derive advantages from the biological characteristics of NPs, including reduced dosage, decreased antagonism, increased absorption rate and improved tissue distribution in animals. The considerable capacity of NPs, especially at small dosages, has been extensively proven in animal nutrition studies for growth efficacy, feed efficacy and health conditions.

NPs of micro‐ and macro‐minerals reliably enhance body weight, average daily increase and feed conversion efficiency (Bąkowski et al. 2018; Reda et al. 2023). Nutritional products are employed to fulfil an animal's dietary requirements, enhance productivity, boost immune function and microbial composition and reduce disease risk. NPs are recognized for their antiviral, antifungal, antibacterial, antiprotozoal and antioxidant qualities, among others. Alternatives to antibiotics that promote growth and health include silver, copper, selenium, zinc and cobalt NPs (CoNPs) (Pineda et al. 2012; Abd El‐Hack et al. 2018; Hidayat et al. 2021; Ouyang et al. 2021; El‐Maddawy et al. 2022).

In animal nutrition, nanotechnology is applied for disease detection and treatment, as well as for the delivery of vitamins, minerals, probiotics and medications (Fesseha et al. 2020). Nano minerals, nano enzymes and other additives are examples of how nanotechnology is utilized in animal feed (Pundir et al. 2015; Marappan Gopi et al. 2017). By lessening the antagonistic impact of bivalent cations, particularly in minute minerals, NPs improve nutrient absorption and are advantageous for the nutrition of cattle and poultry as well as for better feed and supplemental use (Marappan Gopi et al. 2017).

CoNPs are utilized in both technical and medical industries due to their enhanced qualities (Iravani et al. 2020). Research has indicated that CoNPs can induce cell killing (Francis et al. 2021), suggesting their potential as innovative nanomedicines for use in phototherapy, thermotherapy and chemotherapy. Cobalt can enhance protein metabolism and facilitate the creation of enzymes, hence improving immunological function. Cobalt NPs are being utilized as prospective treatment agents for several infectious diseases due to their advantageous properties (Anwar, Chi Fung et al. 2019; Sharma et al. 2020). The CoNPs are recognized for their capacity to produce reactive oxygen species (ROS) that exert their inhibitory activities against certain microbes (Turecka et al. 2018). The information about the incorporation of biological cobalt NPs in poultry diets and their influence on performance and health is limited, and it is hypothesized that the addition of dietary biological nano‐cobalt (BNCo) will have beneficial effects on broiler chickens. The present trial was targeted to measure the antimicrobial properties of BNCo and its influence on growth performance, carcass traits, haematological parameters, renal and hepatic functions, immunological and antioxidant status, lipid profile and digestive enzymes in broiler chickens.

## 2 Materials and Methods

### 2.1 Biosynthesis and Characterization of Green Cobalt NPs

**FIGURE 1:**
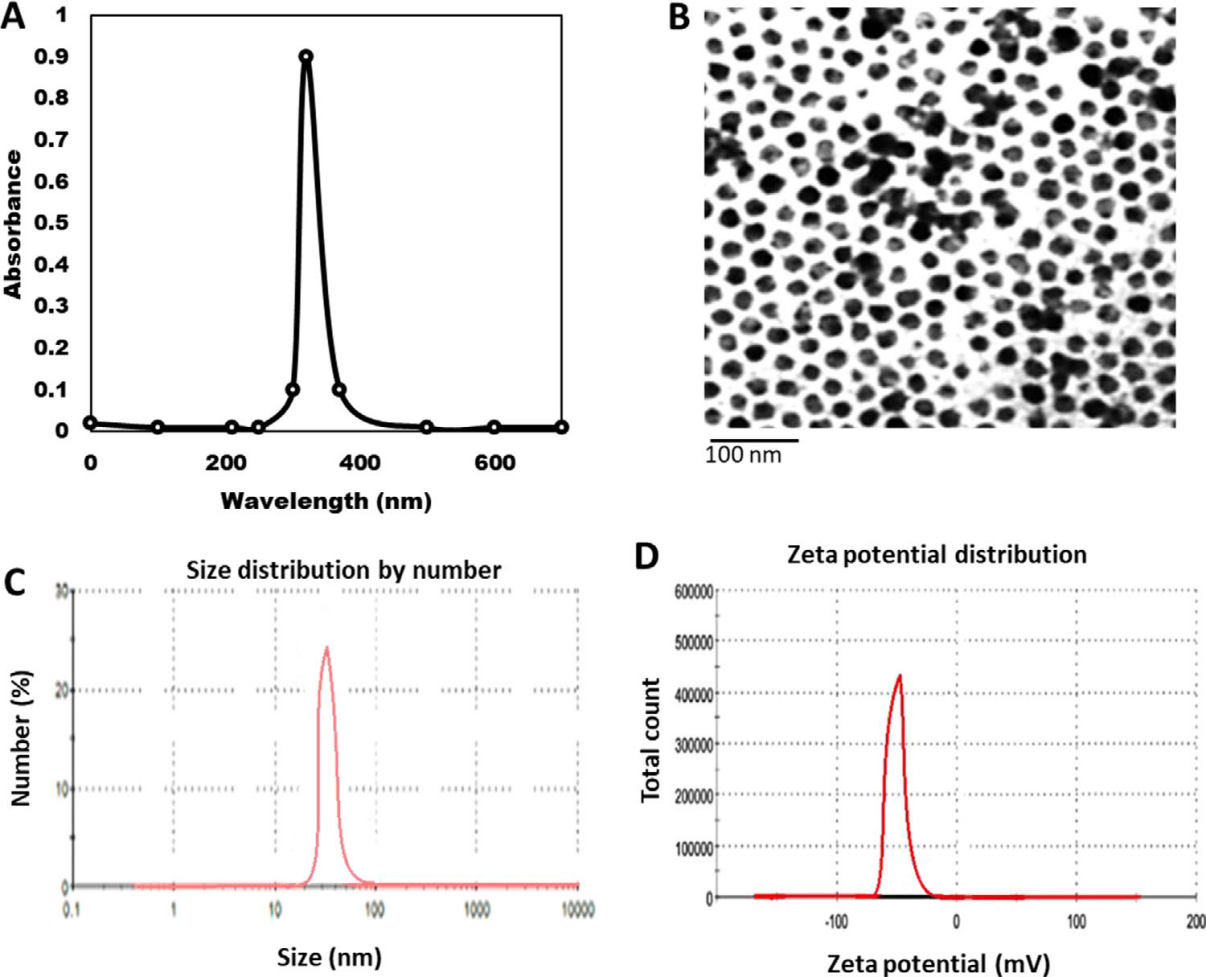
Characterization of green cobalt nanoparticles (A) UV absorbance of BNCo, (B) the shape of BNCo by TEM, (C) the acute size of BNCo by zeta sizer and (D) net charge of BNCo by zeta potential.

The produced green cobalt NPs were characterized using four instruments: ultraviolet (UV), transmission electron microscopy (TEM), Zeta sizer and Zeta potential, with the following results: The spherical BNCos absorb UV at 320 nm, have a TEM of 45–75 nm, have a Zeta potential of −24.6 mV and have a size of 48 nm (Figure 1).

### 2.2 Ethical Approval Statement

The experiment was carried out in the chicken farm within the Faculty of Agriculture, Poultry Department, Zagazig University, Zagazig, Egypt. The trial adheres to the regulations provided by the Zagazig University Ethics Committee regarding the use of experimental animals (Approval No. ZU‐IACUC/2/F/313/2023).

### 2.3 Animals, Design and Diets

A total of 300 Arbour Acre broiler chicks, all unsexed, were separated into 5 treatment groups, each consisting of 60 chicks, at random. Five replications of each group were formed, each containing 12 chicks. The first group followed a control diet free of BNCo, whereas the second, third, fourth and fifth groups received diets fortified with BNCo at concentrations of 100, 200, 300 and 400 ppm, respectively. Composition and chemical analysis of the experimental diets (starter and finisher diets) are displayed in Table 1. BNCo has been characterized and provided by the Department of Agricultural Microbiology, Faculty of Agriculture, Zagazig University, Zagazig, Egypt.

### 2.4 Growth Performance and Carcass Traits

Live body weight (LBW) was recorded at 1, 3 and 6 weeks of age. All growth performance metrics of the birds were documented at three intervals (1–3, 1–6 and 3–6 weeks of age). At 42 days of age, we randomly chose 25 chickens to assess carcass traits. Each bird was weighed and then euthanized. The weight of the heart, liver and gizzard was calculated and stated as a percentage of the LBW before euthanasia.

### 2.5 Blood Metabolites

Five blood samples from each treatment were randomly taken into heparinized test tubes to assess the blood picture parameters: white blood cells (WBCs, 10^3^/µL), red blood cells (RBCs, 10^6^/µL) and haemoglobin (Hb, g/dL). To ascertain the blood biochemical parameters, the residual blood was subjected to centrifugation to obtain plasma. The biochemical components, including protein and its portions, aspartate aminotransferase (AST), alanine aminotransferase (ALT), renal function parameters such as creatinine, urea and uric acid, also triglycerides (TG), total cholesterol (TC) and its subtype as high‐density lipoprotein (HDL), low‐density lipoprotein (LDL) and very‐LDL (VLDL), and immunoglobulins fractions (IgY, IgA and IgM), were analysed using an automatic analyser with commercial kits from Bio‐diagnostic Company (Giza, Egypt) in accordance with the manufacturer's protocol. Superoxide dismutase (SOD), malondialdehyde (MDA), catalase (CAT), total antioxidant capacity (TAC) and reduced glutathione (GSH) levels in plasma were quantified using commercial kits from Biodiagnostic Company (Giza, Egypt).

### 2.6 Digestive Enzymes

The Somogyi (1960) technique was utilized to evaluate amylase activity, whereas the analysis of lipase enzyme adhered to the protocol established by Tietz and Fiereck (1966). The methodologies stated by Lynn and Clevette‐Radford (1984) were utilized to evaluate the action of the protease enzyme.

### 2.7 Statistics

Statistical analyses were conducted utilizing SAS software. The growth metrics, carcass traits, blood profile, blood parameters and digestive enzymes were applied using a one‐way analysis of variance (*p* < 0.05).

**TABLE 1:** Composition and chemical analysis of the experimental diets (starter and finisher diets).

Items	Starter	Finisher
**Ingredients (%)**		
Yellow corn	56.85	61.25
Soybean meal	34.30	32.80
Soybean oil	0.70	2.50
Corn gluten (62% CP)	4.50	0.00
Di‐calcium phosphate	0.20	0.25
Limestone	2.20	2.00
Vit‐min Premix^a^	0.30	0.30
NaCl	0.30	0.30
DL methionine	0.17	0.20
l‐Lysine	0.28	0.25
Choline chloride 60%	0.20	0.15
Total	100	100
**Calculated analysis^b^ (%)**		
CP	23.11	20.07
ME Kcal/kg diet	2913	3107
Ca	1.00	0.97
P (Available)	0.46	0.45
Lysine	1.40	1.29
M + C	0.92	0.82
CF	3.62	3.57
Linoleic acid	1.38	1.47

^a^ Growth vitamin and mineral premix Each 2.5 kg consists of: Vit A, 12,000,000 IU; Vit D3, 2,000,000 IU; Vit E, 10 g; Vit k3 2 g; Vit B1, 1000 mg; Vit B2, 49 g; Vit B6, 105 g; Vit B12, 10 mg; pantothenic acid, 10 g; niacin, 20 g, folic acid, 1000 mg; Biotin, 50 g; choline chloride, 500 mg, Fe, 30 g; Mn, 40 g; Cu, 3 g; Co, 200 mg; Si, 100 mg and Zn, 45 g.

^b^ Calculated according to National Research Council and Subcommittee on Poultry Nutrition (1994).

## 3 Results and Discussion

### 3.1 Growth Performance

The growth performances of broiler chicks as influenced by dietary supplementation with BNCo are illustrated in Table 2. The finding illustrates a significant increase in LBW at 3 and 6 weeks (*p* < 0.05, *p* < 0.01). The third group, treated with 200 ppm BNCo, showed increased results (912.33 and 2233.48 g). Moreover, body weight gain was significantly increased during the 3–6 and 1–6 weeks (*p* < 0.001, *p* < 0.0001), and the third group showed the most pronounced results (62.91 and 58.55 g). However, the feed intake showed no significant variations throughout the trial period: 1–3 weeks, 3–6 weeks, 1–6 weeks. Furthermore, the third group presented significantly better FCR during the 3–6 week period (*p* < 0.01) and the 1–6 week period (*p* < 0.01), with values of 1.97 and 1.78, respectively.

**TABLE 2:** Growth performance of broiler chicks as affected by dietary treatments at 6 weeks of age.

	BNCo (ppm)						
Items	0	100	200	300	400	SEM	*p* value
**Body weight (g)**							
1 week	184.83	185.5	184.17	185.53	184.83	1.538	0.9718
3 week	874.63^c^	902.03^ab^	912.33^a^	910.87^ab^	884.47^bc^	8.150	0.0306
6 week	2047.90^c^	2143.97^b^	2233.48^a^	2074.20^c^	2015.70^c^	19.297	0.0001
**Body weight gain (g/day)**							
1–3 week	49.27^c^	51.18^abc^	52.01^a^	51.81^ab^	49.97^bc^	0.595	0.0358
3–6 week	55.87^c^	59.14^b^	62.91^a^	55.40^c^	53.87^c^	0.941	0.0004
1‐6 week	53.23^c^	55.95^b^	58.55^a^	53.96^c^	52.31^c^	0.524	<0.0001
**Feed intake (g/day)**							
1–3 week	73.61	74.75	74.94	72.86	73.23	0.962	0.5444
3–6 week	123.47	124.86	124.09	128.19	128.3	1.932	0.3099
1–6 week	103.53	104.82	104.43	106.06	106.27	1.361	0.6106
**Feed conversion ratio (g/g)**							
1–3 week	1.49	1.46	1.44	1.41	1.47	0.019	0.1253
3–6 week	2.21^ab^	2.11^bc^	1.97^c^	2.31^a^	2.38^a^	0.047	0.0019
1–6 week	1.95^ab^	1.87^bc^	1.78^c^	1.97^ab^	2.03^a^	0.027	0.0014

*Note*: Means in the same raw with no superscript letters after them or with a common superscript letter following them are not significantly different (*p* < 0.05). Overall treatment *p* value.

Abbreviations: BNCo, biological nano‐cobalt; SEM, standard error means.

Most poultry studies have focused on the effects of supplementing with mineral nanoforms to promote growth and mineral retention (Hossain 2025; Nechitailo et al. 2025). Our result discovered a significant improvement (*p* < 0.05) in birds' body weight, body weight gain and feed conversion ratio, whereas feed intake was influenced non‐significantly. The improvement of BWG in the BNCo‐treated group may be due to the greater bioavailability of Co in the NPs. Our finding aligns with the data of Halle et al. (2011), who demonstrated that treatments with 0.65 mg Co enhanced feed consumption and, consequently, the growth of broilers. The FCR was influenced mainly during the initial 2 weeks by elevated concentrations of vitamin B12 and vitamin B12 combined with BNCo in the feed. With the same result, Ibrahim et al. (2017) demonstrated that the broilers given diets supplemented with nano‐ZnO (110 mg/kg) revealed improvement in BWG and FCR compared to those given diets treated with organic Zn and inorganic ones.

Furthermore, our findings agree with Mohammadi et al. (2015), who noted enhanced growth performance in chickens treated with nano‐zinc‐methionine and nano‐zinc‐max at 80 mg/kg of diet. Conversely, dietary nano‐zinc sulphate decreases growth performance in broiler chicken. Nano‐silver treatments at 4 mg/kg enhanced BWG and optimal FCR in broilers (Elkloub et al. 2015). Andi et al. (2011) confirmed that silver, which acts as an antimicrobial agent against harmful gut microbes, may promote gut health, leading to improved nutrient absorption, as demonstrated by increased body mass, feed intake and feed conversion rate in broiler chickens consuming feed with AgNP.

### 3.2 Carcass Characteristics

The carcass characteristics of broiler chicks influenced by dietary supplementation with BNCo are illustrated in Table 3. The data illustrated a non‐significant variation in carcass characteristics (carcass, liver, gizzard, heart, giblets and dressing) percentage. The second group showed an increased carcass and heart percentage (74.46% and 72%, respectively), whereas the fourth group presented increased liver, giblet and dressing percentages (2.61%, 6.58% and 80.77%), respectively.

**TABLE 3:** Carcass traits of broiler chicks as affected by dietary treatments at 6 weeks of age.

	BNCo (ppm)						
Items (%)	0	100	200	300	400	SEM	*p* value
Carcass	73.03	74.46	74.39	74.19	73.23	0.938	0.7369
Liver	2.37	2.35	2.24	2.61	2.51	0.140	0.4668
Gizzard	3.14	3.17	3.41	3.27	3.28	0.141	0.7213
Heart	0.55	0.72	0.68	0.69	0.59	0.068	0.3779
Giblets	6.06	6.24	6.34	6.58	6.38	0.190	0.5613
Dressing	79.09	80.7	80.73	80.77	79.61	1.077	0.7321

*Note*: Overall treatment *p* value.

Abbreviations: BNCo, biological nano‐cobalt; SEM, standard error means.

The carcass parameters used in this study, which included the broiler's carcass, gizzard, heart, giblets and dressing percentage, did not significantly differ depending on the concentration of Nano‐Co treatments. The adoption of a good, balanced basal diet may be the reason for the lack of notable variations in carcass criteria. After a certain amount of BNCo, carcass features may no longer be improved. Research has demonstrated that the addition of 0.3–0.5 mg/kg of nano‐selenium to feed had no influence on the carcass characteristics and organ weights of broilers, which aligns with our findings (Bakhshalinejad et al. 2019; Ashour et al. 2025). Similarly, Wang et al. (2011) demonstrated that there were no appreciable changes in carcass criteria after supplementing with nano‐selenium at doses of 50–150–300 µg/kg. Moreover, our findings match with the results of Esfahani et al. (2015), who stated that the addition of zinc oxide NPs in a dry chicken diet enhanced carcass traits and the proportional weight of digestive and lymphoid organs in comparison to a wet diet at the starter (1–21) day period.

### 3.3 Haematology

The results showed a significant elevation in Hb and RBCs (*p* < 0.05, *p* < 0.01) in groups supplemented with BNCo, as explained in Table 4. The third group illustrated an elevated level of Hb (8.25 g/dL), whereas the fourth group presented increased numbers of RBCs (4.12 × 10^6^/µL) relative to the control. The BNCo groups presented a significant (*p* = 0.0159) rise in the number of WBCs. The finding reported a significant rise in Hb, RBCs and WBCs in the groups supplemented with BNCo. Our result agrees with Diaz et al. (1994), who stated an elevation in erythrocytes and Hb in broilers treated with a diet of cobalt. Conversely, Kato et al. (2003) reported that the haematocrit, Hb, erythrocytes and leukocyte counts were unaffected by cobalt and vitamin B12 treatment in the diet, whereas erythrocyte and Hb levels were unaffected by cobalt‐only treatment. Cobalt, as the primary cofactor of vitamin B12, is essential for nucleotide combination and can also activate the haematological system in bone marrow, hence enhancing Hb synthesis and increasing RBC production (Chen et al. 2021). The complicated methods by which cobalt stimulates haematopoiesis are summarized as follows: First, as mentioned earlier, cobalt is involved in the metabolism of RNA and blood components as the active centre of vitamin B12, affecting the haematopoietic process (Danzeisen et al. 2020). A lack of vitamin B12 can lead to megaloblastic anaemia because of the suppression of the synthesis of DNA due to reduced pyrimidine and purine availability, resulting in increased (RBCs) size and the increase of substantial, undeveloped precursor cells (megaloblasts) of RBCs in the bone marrow and blood (Tjong et al. 2022).

**TABLE 4:** Haematology of broiler chicks as affected by dietary treatments at 5 weeks of age.

	BNCo (ppm)						
Items	0	100	200	300	400	SEM	*p* value
Haemoglobin	7.67^b^	7.95^ab^	8.25^a^	7.85^b^	7.97^ab^	0.106	0.0404
RBCs	3.31^c^	3.65^bc^	4.00^ab^	4.12^a^	3.79^ab^	0.106	0.0039
WBCs	19.87^b^	22.68^a^	22.49^a^	23.44^a^	23.51^a^	0.623	0.0159

*Note*: Means in the same raw with no superscript letters after them or with a common superscript letter following them are not significantly different (*p* < 0.05). Overall treatment *p* value.

Abbreviations: BNCo, biological nano‐cobalt; RBCs, red blood cells; SEM, standard error means; WBCs, white blood cells.

### 3.4 Liver and Kidney Functions

Hepatic and renal functions of broiler chicks as impacted by dietary supplementation with BNCo are shown in Table 5. The conclusions illustrated a significant rise in TP, ALB and GLOB (*p* < 0.001, *p* < 0.01, *p* < 0.01), and the third group revealed increased levels of TP and ALB (4.47, 2.61 g/dL), whereas the fourth group showed the increased GLOB level (1.96 g/dL) relative to control and other groups. Liver enzymes AST and ALT showed a significant variation (*p* < 0.001, *p* < 0.01) with BNCo treatments, and the third group revealed decreased levels (52.38, 15.30 IU/L) relative to control and other treatments. Kidney function tests (creatinine and urea) illustrated a significant improvement with BNCo treatments (*p* < 0.01, *p* < 0.05), and the third group revealed decreased levels of creatinine and urea (0.72 and 2.34 mg/dL). On the other hand, uric acid levels were not significantly affected by BNCo supplementation (*p* = 0.0953).

**TABLE 5:** Liver and kidney functions of broiler chicks as affected by dietary treatments at 5 weeks of age.

	BNCo (ppm)						
Items	0	100	200	300	400	SEM	*p* value
TP (g/dL)	3.38^b^	4.25^a^	4.47^a^	4.28^a^	4.14^a^	0.116	0.0006
ALB (g/dL)	1.95^c^	2.47^ab^	2.61^a^	2.32^b^	2.52^ab^	0.076	0.0011
GLOB (g/dL)	1.43^c^	1.77^ab^	1.86^a^	1.96^a^	1.62^bc^	0.070	0.0027
A/G (%)	1.36^ab^	1.40^ab^	1.41^ab^	1.18^b^	1.56^a^	0.059	0.0330
AST (IU/L)	75.48^ab^	60.79^cd^	52.38^d^	67.90^bc^	82.18^a^	3.355	0.0008
ALT (IU/L)	20.41^ab^	17.84^bc^	15.30^c^	21.22^ab^	23.49^a^	1.154	0.0048
Creatinine (mg/dL)	1.05^ab^	0.83^bc^	0.72^c^	1.01^ab^	1.22^a^	0.070	0.0061
Uric acid (mg/dL)	7.04	5.16	6.01	6.78	7.41	0.544	0.0953
Urea (mg/dL)	3.51^a^	2.60^bc^	2.34^c^	3.59^a^	3.19^ab^	0.244	0.0206

*Note*: Means in the same raw with no superscript letters after them or with a common superscript letter following them are not significantly different (*p *< 0.05). Overall treatment *p* value.

Abbreviations: A/G, albumin/globulin ratio; Alb, albumin; ALT, alanine aminotransferase; AST, aspartate aminotransferase; BNCo, biological nano‐cobalt; GLOB, globulin; SEM, standard error means; TP, total protein.

The liver function test significantly improved by increasing total protein, albumin and globulin in the blood serum of broiler chicks, whereas AST and ALT serum levels significantly decreased by BNCo supplementation. In the meantime, all treatments with BNCo levels showed a significant drop in serum liver function enzymes (ALT and AST) and renal function tests (creatinine, urea and uric acid). The improved liver and kidney functions seen in broilers may be a result of the complex effects of BNCo, which include antioxidant qualities and metabolic regulation. Our findings agreed with Shokraneh et al. (2020), who confirmed that nano‐selenium significantly reduced the concentrations of AST and ALT. Similarly, Qin et al. (2016) demonstrated that nano‐selenium dietary treatment significantly enhanced the liver and kidneys of rabbits. Notably, ALT and AST enzyme levels can be used to measure liver oxidative damage. In this investigation, feed supplementation with BNCo significantly decreased the serum concentration of creatinine, uric acid, urea, ALT and AST in chickens. These findings were corroborated by Sheiha et al. (2020), who found that the blood serum of Cobb chicken supplemented with nano‐selenium at doses of 0.15, 0.075 and 0.0375 ppm had lower levels of AST, ALT and creatinine. Accordingly, adding nano‐SE to the broiler diet may have contributed to the substantial drop in both ALT and AST, which was determined to be caused by nano‐selenium's major effects on fat metabolism through the thyroid (T3) hormone (Dalia et al. 2017).

### 3.5 Lipid Profile

Table 6 illustrates the lipid profile of broiler chicks influenced by feeding treatments with BNCo. The findings revealed a significant improvement in the lipid profile, and the third group demonstrated a significant (*p* < 0.05, *p* < 0.01) reduction in TC and LDL (150 and 77.74 mg/dL). Furthermore, the fourth group presented a significant (*p* < 0.05, *p* < 0.05, *p* < 0.05) decrease in TG, HDL and VLDL (56.50, 46.94 and 11.30 mg/dL).

**TABLE 6:** Lipid profile of broiler chicks as affected by dietary treatments at 5 weeks of age.

	BNCo (ppm)						
Items	0	100	200	300	400	SEM	*p* value
TC (mg/dL)	168.34^ab^	153.50^bc^	150.00^c^	151.38^c^	172.32^a^	4.735	0.0289
TG (mg/dL)	80.03^a^	64.53^b^	61.69^b^	56.50^b^	69.63^ab^	3.935	0.0189
HDL (mg/dL)	51.26^ab^	53.28^ab^	59.92^a^	46.94^b^	48.45^b^	2.666	0.0490
LDL (mg/dL)	101.07^ab^	87.31^cd^	77.74^d^	93.14^bc^	109.94^a^	3.822	0.0019
VLDL (mg/dL)	16.01^a^	12.91^b^	12.34^b^	11.30^b^	13.93^ab^	0.787	0.0189

*Note*: Means in the same raw with no superscript letters after them or with a common superscript letter following them are not significantly different (*p* < 0.05). Overall treatment *p* value.

Abbreviations: BNCo, biological nano‐cobalt; HDL, high density lipoprotein; LDL, low density lipoprotein; SEM, standard error means; TC, total cholesterol; TG, triglycerides; VLDL, very‐low‐density lipoprotein.

According to this study, broiler lipid profile levels were affected by BNCo dietary supplementation, which reported a substantial drop (*p* < 0.05) in cholesterol, TG, LDL and VLDL at doses of 100, 200 and 300 ppm, respectively. Our findings align with earlier research, particularly that by Mohapatra et al. (2014), who confirmed that broilers given 0.3 mg/kg of nano‐selenium had substantially lower cholesterol and triglyceride concentrations than the control group. Similarly, Saleh and Ebeid (2019) confirmed that adding 0.5 mg/kg of dietary nano‐SE to broiler feeds significantly decreased plasma TG and TC.

### 3.6 Antioxidant Status

The antioxidant condition of broiler chicks as impacted by dietary supplementation with BNCo is illustrated in Table 7. The data presented a significant boost in antioxidant status with BNCo treatments. The second group presented a significant drop (*p* < 0.01) in MDA activity (0.22 nmol/mL) and a significant progress (*p* < 0.01) in SOD activity (0.58 U/mL). Furthermore, the third group showed a significant boost (*p* < 0.05, *p* < 0.01, *p* < 0.001) in CAT, TAC and GSH (0.52 mg/dL, 0.57 ng/mL, 0.72 mg/dL), respectively, compared to the control and other treatments.

**TABLE 7:** Antioxidants of broiler chicks as affected by dietary treatments at 6 weeks of age.

	BNCo (ppm)						
Items	0	100	200	300	400	SEM	*p* value
MDA (nmol/mL)	0.42^a^	0.22^b^	0.26^b^	0.23^b^	0.49^a^	0.042	0.0035
SOD (U/mL)	0.31^b^	0.58^a^	0.56^a^	0.56^a^	0.35^b^	0.042	0.0023
CAT (mg/dL)	0.23^b^	0.47^a^	0.52^a^	0.34^ab^	0.36^ab^	0.053	0.0273
TAC (ng/mL)	0.27^c^	0.54^a^	0.57^a^	0.38^bc^	0.43^ab^	0.041	0.0046
GSH (mg/dL)	0.22^c^	0.57^ab^	0.72^a^	0.52^b^	0.31^c^	0.047	0.0002

*Note*: Means in the same raw with no superscript letters after them or with a common superscript letter following them are not significantly different (*p* < 0.05). Overall treatment *p* value.

Abbreviations: BNCo, biological nano‐cobalt; CAT, catalase; GPX, glutathione peroxidase; GSH, reduced glutathione; MDA, malondialdehyde; SEM, standard error means; SOD, superoxide dismutase; TAC, total antioxidant capacity.

The antioxidant status was improved significantly with BNCo supplementation. These outcomes align with Scott et al. (2016) who stated that CoNPs can dramatically boost the creation of lipid peroxidation, and ROS, and boost caspase‐3 enzymes to protect the cells from oxidation. In addition to their average TAC and total reducing impact, the BNCo established exceptional radical scavenging potential (Matinise et al. 2018). Shahzadi et al. (2019) also confirmed that bioinspired CoNPs have radical scavenging activity; they also noted that the antioxidant activity and scavenging power are dosage dependent, meaning that as activity rises, so does CoNP concentration. The green‐synthesized NPs exhibited significant free radical‐scavenging action and yielded remarkable results (Hou et al. 2020). Similarly, it has been observed that cobalt oxide NPs produced from *Sesbania sesban* extract have lower DPPH radical scavenging efficacy than silver and copper oxide NPs (Ghadi et al. 2018). Moreover, Elkhateeb et al. (2024) confirmed that MDA significantly dropped as nano‐Se concentration increased to 0.4 mg/kg, and the TAC provided a substantial linear rise with elevation of nano‐selenium concentration at 0.4 mg/kg. Moreover, Hafez et al. (2020) reported that broiler hens' antioxidant status was improved by adding zinc oxide NPs at 40 or 80 mg/kg to their feed, as shown by amplified SOD and CAT action and diminished MDA levels. Zhao et al. (2014) confirmed that the group administered 20 mg/kg nano‐ZnO exhibited a reduction in serum and liver MDA concentration, enhanced TAO action in both blood and liver tissue and elevated serum CAT action.

Additionally, Zhao et al. (2014) confirmed that feeds supplemented with 60 or 100 mg Zn/kg from ZnO NP had increased action of serum CAT and serum and liver SOD. MDA is typically employed as an oxidative stress indicator (Mateos et al. 2005). The lower MDA in our study following Zn supplementation was consistent with earlier research (Liu et al. 2015).

### 3.7 Immunity

The weight of immunological organs (spleen, thymus and bursa of Fabricius) influenced by BNCo treatments is presented in Table 8. The findings demonstrated a significant (*p* < 0.01, *p* < 0.05) increase in spleen and thymus gland weight percentage, and the third group presented an increased weight percentage (0.15% and 0.60%). In contrast, the second group presented a nonsignificant increase in the bursa of Fabricius weight percentage (0.34%) relative to the control and other treated groups.

**TABLE 8:** Immune organs of broiler chicks as affected by dietary treatments at 6 weeks of age.

	BNCo (ppm)						
Items (%)	0	100	200	300	400	SEM	*p* value
Spleen	0.08^c^	0.13^a^	0.15^a^	0.12^ab^	0.09^bc^	0.01	0.0040
Thymus	0.39^c^	0.55^ab^	0.60^a^	0.45^bc^	0.52^ab^	0.038	0.0262
Bursa of fabricius	0.27	0.34	0.32	0.29	0.26	0.032	0.5158

*Note*: Means in the same raw with no superscript letters after them or with a common superscript letter following them are not significantly different (*p* < 0.05). Overall treatment *p* value.

Abbreviations: BNCo, biological nano‐cobalt; SEM, standard error means.

The immune organs’ weight, especially the spleen, thymus and bursa Fabricius, significantly boosted (*p* < 0.05) with BNCo supplementation to the broilers’ diet. Our results agreed with Grodzik and Sawosz (2006), who confirmed the impact of silver NPs at 10 ppm in embryonic bursa Fabricius and development and found no discernible influence on chicken growth but a decrease in follicle size and quantity. Ahmadi et al. (2010) examined the shifts in the proportional weight of the bursa following the supplementation of Ag–NPs at 20, 40 and 60 ppm concentrations, and there were fewer and smaller follicles. This could be because AgNPs’ antibacterial qualities impact the gut's microbial populations. Ag–NPs undoubtedly slow the development of anaerobic microbes and transfer available oxygen. Therefore, the growth of the bursa of Fabricius is negatively impacted.

On the other hand, the destiny of Ag–NPs in the host may depend on their capacity to elicit an immunological response when they are recognized spontaneously or when immune recognition is absent. The inflammatory reaction by NPs may impact the immunological status and the stability of T helper 1 and T helper 2 cells. According to the findings of Matsumura et al. (2003), a similar impact of silver zeolite may be caused by Ag absorption into bacterial cells upon contact, which impairs cellular processes and causes cell destruction. However, it may be described by the production of reactive oxygen molecules, which prevent cells from functioning properly. It makes sense that microorganisms in the gastrointestinal system are required for the bursa to grow and mature in healthy broilers. The antiviral effectiveness of an Ag–NP solution against the virus that causes infectious bursal disease in chicken embryos was documented in another investigation (Pangestika and Ernawati 2017).

The immunological status of broiler chicks as influenced by dietary supplementation with BNCo is demonstrated in Table 9. The results presented a significant boost in immunity parameters, and the third group revealed a significant increase (*p* < 0.01, *p* < 0.05, *p* < 0.05) in IgA, IgY and lysozymes (0.72, 1.04 and 0.55 mg/dL) relative to the control and other treatments. The second group illustrated a significant boost (*p* < 0.001) in IgM (0.90 mg/dL) relative to control and other treatments. Furthermore, the second group presented a significant increase (*p* < 0.01) in complement 3 (52.58 mg/dL). Additionally, the third group presented a significant increase (*p* < 0.05) in lysozyme levels (0.55 mg/dL) compared to the control and other treatments.

The immunological status was positively affected by BNCo treatment, particularly for IgA, IgY and IgM; additionally, complement 3 and lysozyme were also positively affected. IgM and IgG levels do not seem to be impacted by Ag–NPs (Pineda et al. 2012). The immune system, which is essential to good health and connected to all physiological systems, guards the host against infections (Gao et al. 2014) and continuously monitors native cells that could be dangerous, such as tumour‐forming cells (Pan Na et al. 2011). According to reports, metals are crucially involved in controlling the host's defence against invasive pathogens as well as the innate immune system's perception of them (Anwar, Awais et al. 2019), indicating that metals are involved in controlling the immune system's resistance to infection. A recent study has provided a new medical approach that utilizes metal‐based composites for disease treatment, revealing that these compounds may regulate autophagy, a crucial host immune response (Sahoo et al. 2014).

### 3.8 Digestive Enzymes

The impact of dietary treatments with BNCo on the digestive enzymes of broiler chicks is presented in Table 10. The outcomes presented a significant enhancement in digestive enzymes with BNCo treatments, and the third group revealed a significant increase (*p* < 0.01, *p* < 0.001) in amylase and protease enzymes (182.10 and 3.17 μ/L) in relation to control and other treatments. The second group presented a significant increase (*p* < 0.01) in lipase enzyme (47.34 μ/L) compared to the control and other treatments.

**TABLE 9:** Immunity and antioxidants of broiler chicks as affected by dietary treatments at 6 weeks of age.

	BNCo (ppm)						
Items	0	100	200	300	400	SEM	*p* value
IgA (mg/dL)	0.41^c^	0.59^ab^	0.72^a^	0.44^c^	0.49^bc^	0.040	0.0018
IgM (mg/dL)	0.56^b^	0.90^a^	0.79^a^	0.86^a^	0.53^b^	0.045	0.0004
IgY (mg/dL)	0.73^c^	0.94^ab^	1.04^a^	0.96^ab^	0.84^bc^	0.055	0.0235
C3 (mg/dL)	32.23^c^	52.58^a^	52.03^a^	45.84^ab^	36.98^bc^	2.733	0.0014
Lysozyme (mg/dL)	0.39^c^	0.48^ab^	0.55^a^	0.45^bc^	0.49^ab^	0.024	0.0118

*Note*: Means in the same raw with no superscript letters after them or with a common superscript letter following them are not significantly different (*p* < 0.05). Overall treatment *p* value.

Abbreviations: BNCo, biological nano‐cobalt; IgY and M, immunoglobulin Y; SEM, standard error means.

**TABLE 10:** Digestive enzymes of broiler chicks as affected by dietary treatments at 6 weeks of age.

	BNCo (ppm)						
Items	0	100	200	300	400	SEM	*p* value
Amylase (μ/L)	143.55^bc^	163.14^ab^	182.10^a^	153.26^bc^	136.90^c^	6.815	0.0077
Lipase (μ/L)	21.77^c^	47.34^a^	42.22^ab^	30.01^bc^	23.11^c^	3.800	0.0040
Protease (μ/L)	0.38^d^	2.59^ab^	3.17^a^	1.73^bc^	0.99^cd^	0.292	0.0010

*Note*: Means in the same raw with no superscript letters after them or with a common superscript letter following them are not significantly different (*p* < 0.05). Overall treatment *p* value.

Abbreviations: BNCo, biological nano‐cobalt; SEM, standard error means.

The digestive enzymes, such as amylase, lipase and protease, significantly improved, and these discoveries align with the conclusions of Brugger et al. (2016), who illustrated that on Day 35, the HME‐Zn supplementation significantly raised the amylase activity (*p* < 0.05). In zinc metabolism, exocrine glands like the pancreatic bile regulate zinc homeostasis through the pancreatic digestive enzymes to be secreted by the GIT. Similar to this study, the activities of broiler pancreatic amylase, lipase and trypsin were elevated in diets treated with organic zinc sources that promoted zinc absorption (Hu et al. 2022). Nonetheless, Park et al. (2015) revealed that treatments containing 100, 2500 and 100 mg/kg of coated zinc oxide did not differ in their effects on amylase and the action of trypsin. Nevertheless, the chicks’ pancreas's amylase, lipase and trypsin activity significantly decreased as a result of the excessive additional zinc (2000 mg/kg) (Lü et al. 1988).

In conclusion, broiler chickens given BNCo supplements had better growth performance, carcass traits and antioxidant activity. The beneficial hypolipidemic effects were demonstrated by dietary BNCo supplementation, which reduced serum triglyceride and cholesterol levels. BNCo supplementation at 200 ppm/kg resulted in the greatest overall performance of broilers, suggesting that it could be a novel and useful addition to broiler diets. To better understand how BNCo works as an antioxidant, antibacterial and immune system enhancer in broilers' internal organs, as well as how it affects the sustainability of productive performance, further research is required.

## Author Contributions

Fayiz M. Reda, Abdullah S. Alawam, Hemat K. Mahmoud, Mohamed T. El‐Saadony, Ayman S. Salah, Hassan A. Rudayni, Ahmed A. Allam, Karima El‐Naggar, Mahmoud Alagawany and Most Khairunnesa were involved in conceptualization, data curation, formal analysis, investigation, methodology, resources, validation, visualization, roles/writing of the original draft, writing review and editing.

## Conflicts of Interest

The authors declare no conflicts of interest.

## Peer Review

The peer review history for this article is available at https://publons.com/publon/10.1002/vms3.70508.

## Data Availability

The data in this study can be obtained upon request from the relevant authors.
